# Residence Time Structures Microbial Communities Through Niche Partitioning

**DOI:** 10.1111/ele.70093

**Published:** 2025-02-26

**Authors:** Emmi A. Mueller, Jay T. Lennon

**Affiliations:** ^1^ Department of Biology Indiana University Bloomington Indiana USA

**Keywords:** chemostat, community assembly, consumer‐resource, immigration, niche partitioning

## Abstract

Much of life on Earth is at the mercy of currents and flow. Residence time (*τ*) estimates how long organisms and resources remain in a system based on the ratio of volume (*V*) to flow rate (*Q*). Short *τ* should promote immigration but limit species establishment, while long *τ* should favour species that survive on limited resources. Theory suggests these opposing forces shape the abundance, diversity and function of flowing systems. We experimentally tested how residence time affects a lake microbial community by exposing chemostats to a *τ* gradient spanning seven orders of magnitude. Microbial abundance, richness and evenness increased non‐linearly with *τ*, while functions like productivity and resource consumption declined. Taxa formed distinct clusters of short‐ and long‐*τ* specialists consistent with niche partitioning. Our findings demonstrate that residence time drives biodiversity and community function in flowing habitats that are commonly found in environmental, engineered and host‐associated ecosystems.

## Graphical abstract

Much of life on earth is at the mercy of currents and flow. Residence time (*τ*) estimates how long organisms and resources remain in a system based on the ratio of volume (*V*) to flow rate (*Q*). We tested predictions about how residence time affects abundance, diversity and function of microbial communities, leading to the emergence of niche partitioning.

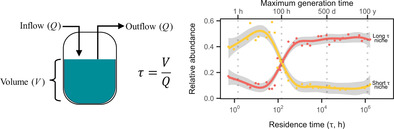

## 1 Introduction

In nature, physical forces influence the movement of resources and organisms in complex ways that determine the structure and function of communities (Ambrosetti et al. 2003; Bell et al. 2002; Kadlec 1994; Kashyap et al. 2013). Residence time (*τ*) captures some of this physical complexity by estimating the average amount of time passively moving molecules and organisms spend in a system. Operationally, it is quantified as the ratio of volume (*V*) to flow rate (*Q*) of a simple, well‐mixed system (Nauman 2008). Residence time has long been used to characterise the growth and metabolism of planktonic organisms in continuous flow reactors (i.e., chemostats). For example, residence time theory predicts that productivity (*P*) and growth rate (*μ*) of a population are both maximised when the dilution rate (1/*τ*) of a system is equal to the maximum growth rate (*μ*_max_) of the population (Smith and Waltman 1995). Many of the core principles derived from residence time form the basis of other ecological frameworks, including consumer‐resource theory (Grover 1997; Sommer 1992; Tilman 1982).

Despite differences in scale and complexity, shared physical processes underlie the population‐ and community‐level patterns that might emerge in systems with varying residence times. Specifically, two opposing forces influence survival and reproduction along a residence time gradient. When *τ* is short, there may be high rates of immigration from the regional pool of species along with an elevated resource supply that should favour fast‐growing species. Although some individuals are likely to be washed out of the system at high flow rates, slower‐growing individuals may resist this source of mortality through active dispersal against currents or attachment to surfaces (Stemmons and Smith 2000). In contrast, when *τ* is long, the resource supply rate may be insufficient to meet the maintenance requirements for some species due to resource limitation. Under such conditions, individuals that do not invest in persistence strategies, such as slow growth, efficient resource consumption or decreased metabolism, are likely to starve (Lennon and Jones 2011; Shoemaker et al. 2021). Species may contend with the opposing forces of washout and resource limitation in various ways depending on their traits and assembly processes (Locey and Lennon 2019), which could potentially lead to niche partitioning along the residence time axis, both within environments with fluctuating residence times and between environments of different residence times.

While residence time is a powerful framework that is well‐supported by empirical observations (Amster et al. 2020; Smith and Waltman 1995), it was originally developed for idealised conditions that are commonly violated in nature. It typically assumes no immigration, constant population growth and uniformly mixed conditions that support simple communities consisting of just a few species (Harmand et al. 2017). Recently, efforts have been made to incorporate additional complexity into residence time theory using stochastic individual‐based models (IBMs). Across combinations of volumes and flow rates, the models identify potential constraints while making predictions that organismal abundance (*N*), species richness (*S*) and productivity should peak at intermediate residence time (Locey and Lennon 2019). Although these models capture multiplicative interactions among species and resources, they are still relatively simple compared to the complexities that can emerge in real‐world systems.

Here, we experimentally evaluated the effects of residence time on community structure and function in continuous flow reactors (i.e., chemostats) exposed to a residence time gradient spanning seven orders of magnitude. We inoculated these chemostats with a natural assemblage of lake microorganisms and characterised patterns in abundance and productivity that developed. In addition to assessing community assembly and resource consumption, we tested for evidence of niche partitioning and evaluated the degree to which microbial taxa are specialised along the residence time gradient. Together, our findings contribute to a framework that aims to explain the maintenance of biodiversity in ecosystems subject to forces of physical turnover.

## 2 Methods

### 2.1 Experimental Design

**FIGURE 1:**
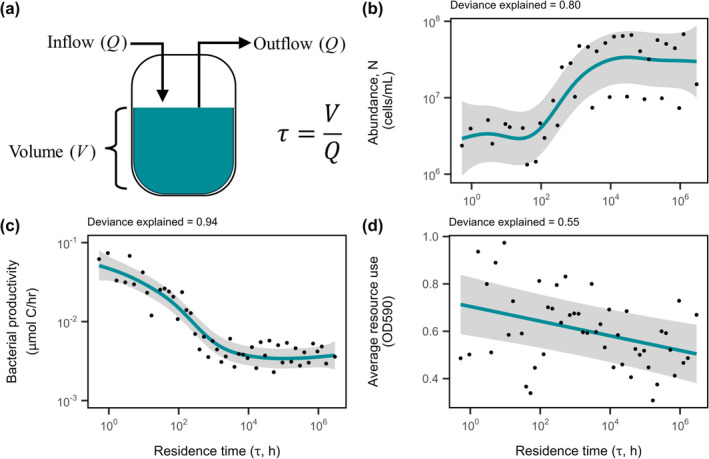
Abundance, productivity and resource consumption of microbial communities along a residence time gradient. (a) We established a gradient of residence times by adjusting the flow rate (*Q*) of lake‐water medium into well‐mixed, fixed‐volume (*V*) bioreactors (chemostats) that were inoculated with an aquatic microbial community. Generalised additive models (GAMs) describing the effect of residence time on (b) abundance (*N*), measured as cells/mL, (c) productivity, measured as the rate of biomass production (μmol C/h) through a tritiated leucine assay and (d) average resource consumption for each community measured via BioLog EcoPlates. Lines and shading represent fits and 95% CIs of GAM regressions, respectively. The percentage of deviance explained by the model is reported above each plot (Table 1).

We experimentally manipulated residence time across an array (*n* = 49) of chemostats (Figure 1a). We supplied each chemostat with twice‐autoclaved lake water as medium, which was collected from University Lake, a meso‐eutrophic reservoir located in Griffy Woods, Bloomington, Indiana, USA (39.189° N, 86.503° W) with a residence time of approximately 124 days (see Supporting Information Methods). At the beginning of the experiment, we added a 1.5 mL inoculum from University Lake to 38.5 mL autoclaved lake‐water medium in each chemostat. This inoculum consisted of a 2:1 (vol:vol) mixture of freshly collected unfiltered lake water (10^7^ cells/mL) and a dilute suspension of lake sediments (10^9^ cells/mL). We collected samples for the inoculum from a site located 125 m upstream of the dam, which has previously been shown to contain autochthonous (lake) and allochthonous (soil) bacteria (Wisnoski et al. 2020) spanning a range of growth rates (Wisnoski and Lennon 2021). Following inoculation, we allowed the chemostats to equilibrate for 24 h at 25°C before initiating the experiment (time = Day 0). We held the volume (*V*) of each chemostat constant (40 mL) while adjusting flow rate (*Q*), using either peristaltic pumps or manual pipetting depending on the residence time (Figure S1; see Supporting Information Methods). We ran the experiment across four experimental blocks in an unreplicated regression design to generate a residence time gradient ranging from approximately 30 min to 330 years, which corresponded to dilution rates (1/*τ*) of 1.9/h to 0.003/year (see Supporting Information Methods). Chemostats were incubated at 25°C and homogenised with a magnetic stir bar. Because rates of resource input and immigration are often proportional to volume and flow rate, we reintroduced microorganisms from a sediment sample at a residence‐time‐dependent concentration, which amounted to 1% of the abundance that turned over each day. The sediment sample used for reintroduction was the same as that used for inoculation. To approximate in situ conditions and minimise compositional changes, we stored this sediment sample at 4°C throughout the experiment. At the end of the experiment (time = Day 20), we destructively sampled the chemostats to measure microbial community structure and function. Although it is unlikely that long‐residence‐time chemostats would reach a steady state within 20 days, this standardised duration allowed us to infer the effects of residence time on microbial community abundance, diversity and function in our experiment.

### 2.2 Microbial Abundance

We measured microbial abundance using flow cytometry. After diluting samples 1:100 in phosphate buffered saline (PBS), we stained samples with 1 μL *Bac*Light RedoxSensor Green (RSG, Invitrogen, Waltham, MA, USA, excitation/emission: 490/520 nm), a cell permeable electron transport chain activity indicator, per 1 mL of sample. We left a replicate set of samples unstained, incubating all samples for 15 min in complete darkness at 37°C. We fixed all samples with 25 μL of 25% glutaraldehyde and incubated them again for 30 min in complete darkness at 25°C. Fixed cells were stored in the dark at 4°C until they were run on a NovoCyte 3000VYB (Agilent, Santa Clara, CA, USA) with a 50 mW laser‐emitting light at 488 nm and a 530 nm filter measuring green fluorescence from the RSG (see Supporting Information Methods). We processed the data with NovoExpress software (version 1.4.1), using electronic gating (Figure S2), calculating absolute abundance as:

(1) $$\text{Absolute count}=\frac{\text{events in gate}\times \text{dilution factor}}{\text{sample acquisition volume}}$$

### 2.3 Bacterial Productivity

We measured bacterial productivity (BP) in each chemostat using a tritiated (^3^H) leucine assay with four 1.5 mL sample technical replicates (Smith and Azam 1992). We added ^3^H‐leucine (161 Ci/mmol; half‐life of 12.346 years) to a final concentration of 50 nM and immediately stopped leucine incorporation in one replicate sample (kill‐control) by adding 300 μL of 3 mM trichloroacetic acid (TCA). After 60 min of incubation for the remaining live samples (*n* = 3), we stopped the reactions with 300 μL of 3 mM TCA, removed unincorporated ^3^H‐leucine, washed samples with 1.5 mL of 0.3 mM TCA and resuspended cell pellets in scintillation cocktail. The amount of radioactive leucine incorporated into bacterial proteins was measured with a Tri‐Carb 2100TR Liquid Scintillation Counter (machine efficiency: 63.9%; Packard Instrument Company, Meriden, CT, USA). Measured counts per minute (CPMs) were converted to incorporation rates of μmol C/h using estimates of cellular C per protein and the fraction of leucine in protein and averaged across technical replicates for downstream analyses (Kirchman 2001; Muscarella et al. 2020).

### 2.4 Resource Consumption

We estimated changes in the resource consumption profile of microbial communities along the residence time gradient with Biolog EcoPlates (Biolog, Hayward, CA, USA). Ecoplates contain 31 carbon sources along with a tetrazolium dye. Carbon substrate consumption is estimated by the colour change of tetrazolium dye, which is reduced in the presence of NADH, a product of cellular metabolism (Garland and Mills 1991). The 31 carbon sources can be grouped into seven higher‐level categories: carboxylic acids, polymers, carbohydrates, amino acids, amines, esters and phosphorylated carbon sources. We determined carbon consumption for each chemostat by inoculating each EcoPlate well with a 150 μL chemostat sample from the end of the experiment (Day 20), wrapping the plates in parafilm, then measuring OD (590 nm) after a 48‐h incubation at 25°C and subtracting the water blanks. Response values > 0.125 OD (590 nm) were counted as positive for consumption of a given carbon source (Garland 1997). We then calculated the community resource consumption at each residence time by calculating the mean consumption of all carbon sources, each in triplicate, on the EcoPlate. We used principal coordinates analyses (PCoA) to characterise residence time effects on resource consumption at 48 h. For our PCoAs, we used the Bray–Curtis dissimilarity metric from the ‘vegan’ package (version 2.6‐4) in R (Oksanen et al. 2012).

### 2.5 Microbial Communities

#### 2.5.1 Diversity

We measured microbial diversity using high‐throughput 16S rRNA gene sequencing (Locey et al. 2020; Wisnoski et al. 2020). Detailed methods describing DNA extraction, PCR conditions, sequencing and assembly can be found in the Supplemental Methods. After binning sequences with > 97% similarity to create operational taxonomic units (OTUs), we quantified richness (*S*) as the observed number of taxa after rarefaction. We calculated evenness (*E*) using Simpson's index (*D* ^−1^/*S*) where *S* is taxon richness and *D* ^−1^ is the inverse of Simpson's diversity (Maurer and McGill 2011). We characterised the effects of residence time on univariate measures of diversity using generalised additive models (described below) and on multivariate measures of diversity with PCoAs (described above).

#### 2.5.2 Niche Partitioning

We tested whether there was a non‐random degree of overlap in the relative abundance of microbial taxa along the residence time gradient using the Pianka index, a common measure of niche partitioning (Piana and Marsden 2012)

(2) $$\begin{array}{c} O_{12}=\frac{\sum_{i=1}^{n}\left(P_{i2}\times P_{i1}\right)}{\sqrt{\sum_{i=1}^{n}{P_{2i}}^{2}\times \sum_{i=1}^{n}{P_{1i}}^{2}}} \end{array}$$

where *P*_*ij*_ is the relative abundance of taxon *j* at residence time *i* and *n* is the total number of residence times, where values close to 1 reflect similar use of the residence time gradient and values close to 0 reflect minimal overlap in use (Pianka 1973). We first calculated observed mean gradient use overlap of taxa found in > 70% of sites with the ‘niche.overlap’ function in the ‘spaa’ R package (version 0.2.2) using method ‘pianka’ (Pianka 1973). This cutoff was chosen to capture niche overlap among the most dominant taxa in our chemostat communities. To test for significance, we then compared observed overlap in relative abundance of taxa with values generated from null models. We generated two sets of 100 randomised chemostat × taxa matrices. In one set, all taxon abundances were drawn from a uniform distribution [0,1]. In the second set, observed zero abundances were preserved but all other elements of the matrix were drawn from a uniform distribution [0,1] (Gotelli and Graves 1996; Winemiller and Pianka 1990).

Next, we identified niches of taxa that were non‐randomly positioned along the residence time gradient. We first calculated the Pearson's correlation coefficient of the relative abundance patterns of taxa across the *τ* gradient for each pair of taxa. From those values, we generated a distance matrix (where $\text{distance}=\frac{1-r}{2}$) and performed *k*‐means clustering by minimising within‐cluster variance (‘kmeans’ function from the ‘stats’ R package, version 4.3.2). To determine the appropriate number of niches, we plotted within groups sum of squares by number of clusters. From this plot, we identified the number of niches where within group sums of squares was minimised without over‐splitting (*n* = 5). We then calculated a combined relative abundance for each niche of taxa at each residence time to visualise the combined relative abundance pattern of these niches. To determine if the niches identified were phylogenetically clustered or randomly dispersed, we calculated phylogenetic dispersion (*D*) of the two major niches using the ‘phylo.d’ function in the ‘caper’ R package (version 1.0.3; Fritz and Purvis 2010) where *D* close to 0 indicates phylogenetic clustering and *D* close to 1 indicates Brownian trait dispersion. We then calculated the Cohen's *d* of these phylogenetic *D* values compared to a null distribution of *D* values generated by 1000 permutations of niche identity under random phylogenetic structure.

### 2.6 Generalised Additive Models

We statistically modelled the effect of residence time on univariate microbial responses using generalised additive models (GAMs). GAMs are linear models where the response variable depends on smoothed functions of predictor variables, allowing for non‐linear responses. We chose GAMs because, without having any a priori expectation of the predictive functions for our data, they accommodate flexible smoothing while still allowing interpretability as each predictive variable is encoded in the model. We used GAMs (‘gam’ function from the ‘mgcv’ R package, version 1.9‐1) to fit bacterial abundance, bacterial productivity, resource consumption, taxon richness, evenness and relative abundance of niches as a function of *τ* using thin plate regression splines as the basis function (*s* (*τ*)) with a smoothing parameter (*λ*) estimated by restricted maximum likelihood (REML) (Wood 2017). We did not restrict the dimension of the basis used for the smoothing term (*k*). We report GAM *p*‐values (*α* = 0.05) from a modified Wald test of whether the coefficient of the smoothed term is equal to zero (Wood 2013). We also report deviance explained for each GAM. When the experimental block was significant, we coded it with a random effect basis function (experimental block, *n* = 4, *s* (Set, bs = ‘re’)). Models were selected based on a conditional Akaike information criterion (AIC) which applies a correction based on the effective degrees of freedom (edf) of the model. All statistical analyses were performed in R (version 4.3.2; R Core Team 2022).

## 3 Results

### 3.1 Longer Residence Times Increased Abundance but Reduced Productivity

At the shortest residence times, bacterial abundance (*N*) was lower than inoculation densities, consistent with regulation by washout. Bacterial abundance increased nonlinearly with increasing residence time, plateauing at ~3 × 10^7^ cells/mL (Table 1; Figure 1b, *F*_4.69_ = 17.0, *p* < 0.0001). In contrast, community bacterial productivity (BP) declined nonlinearly with increasing residence time, flattening out to a baseline of approximately 0.003 μmol C/h (Table 1; Figure 1c, *F*_5.04_ = 97.0, *p* < 0.0001).

**TABLE 1:** Effects of residence time on community structure.

Fit model	Deviance explained	Model *p‐*value	Term	Edf	*F‐*statistic	*p‐*value	REML
Abundance	0.803	0.0001	*s* (*τ*)	4.69	17.04	< 0.0001	13.2
			*s* (Set, bs = re)	1.78	9.70	0.0002	
Bacterial productivity	0.940	< 0.0001	*s* (*τ*)	5.04	96.97	< 0.0001	−22.4
			*s* (Set, bs = re)	2.67	8.319	< 0.0001	
Average resource use	0.547	0.0003	*s* (*τ*)	1.00	13.00	0.0007	−29.6
			*s* (Set, bs = re)	2.76	12.69	< 0.0001	
Resource PCoA Axis 1	0.400	0.0007	*s* (*τ*)	3.66	5.45	0.0007	−37.5
Resource PCoA Axis 2	0.680	0.0002	*s* (*τ*)	6.39	8.54	< 0.0001	−49.7
			*s* (Set, bs = re)	2.17	2.84	0.0144	
Species richness	0.837	0.0093	*s* (*τ*)	5.06	18.23	< 0.0001	257.7
			*s* (Set, bs = re)	2.29	2.95	0.0186	
Species evenness	0.722	< 0.0001	*s* (*τ*)	4.97	11.51	< 0.0001	−88.3
Composition PCoA Axis 1	0.979	0.0055	*s* (*τ*)	7.88	119.01	< 0.0001	−38.7
			*s* (Set, bs = re)	2.41	3.67	0.0110	
Composition PCoA Axis 2	0.786	< 0.0001	*s* (*τ*)	5.35	15.66	< 0.0001	−40.9
Long‐*τ* Niche	0.937	< 0.0001	*s* (*τ*)	6.91	49.65	< 0.0001	−38.7
Short‐*τ* Niche	0.594	0.0007	*s* (*τ*)	5.68	5.22	0.0007	−82.5

*Note:* Response variables for each generalised additive model (Fit model) are shown with deviance explained and *p*‐value for the total model (Model *p*‐Value). Each smoothed term (Term) has an effective degree of freedom (edf) and *F*‐value (*F*‐statistic). Significance of each smoothed term (Term) was determined from term *p*‐values (*p*‐value) (*α* = 0.05). Restricted maximum likelihood values (REML) for each model are shown.

### 3.2 Residence Time Altered Resource Consumption

**FIGURE 2:**
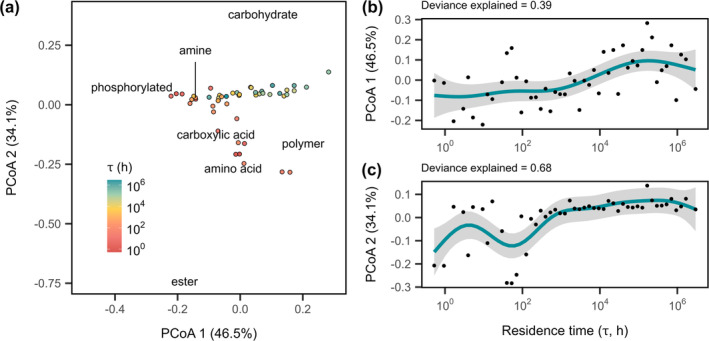
Resource use changed with residence time. (a) Principal coordinate analysis (PCoA) plot showing the composition of resource use of 31 carbon sources of chemostat microbial communities at the end of 20 days of residence time treatment. Distances between circles represent dissimilarity between resource use profiles. Points are coloured by *τ*. Average PCoA resource scores for each resource type are also shown on the plot. Generalised additive models (GAMs) show the effect of residence time on microbial community resource use along the first two axes of the principal coordinate analysis, which account for (b) 47% and (c) 34%, respectively. Lines and shading represent fits and 95% CIs of GAM regressions, respectively. The percentage of deviance explained by the model is reported above each plot (Table 1).

When averaged across all carbon substrates, resource consumption decreased near‐linearly with increasing residence time (Figure 1d; Table 1, *F*_1.00_ = 13.0, *p* = 0.0007). When examined individually, resource consumption of 16 of the 31 carbon substrates changed with residence time (Table S1; Figure S3). Only one (L‐threonine) increased, while the other 15 decreased at long residence times (see Supporting Information Results). In addition, residence time affected the composition of consumed resources. When examined with a principal coordinates analysis (PCoA), the first two axes accounted for 81% of the total variation in consumed resources (Figure 2a). The site scores from the first two axes of the PCoA both increased non‐linearly with increasing residence time, shifting from more ester and amino acid‐based resource use to more carbohydrate‐based resource use (Table 1; Figure 2b, PCoA 1, *F*_3.66_ = 5.4, *p* = 0.0007; Figure 2c, PCoA 2, *F*_6.40_ = 8.5, *p* < 0.0001).

### 3.3 Residence Time Altered Microbial Diversity

**FIGURE 3:**
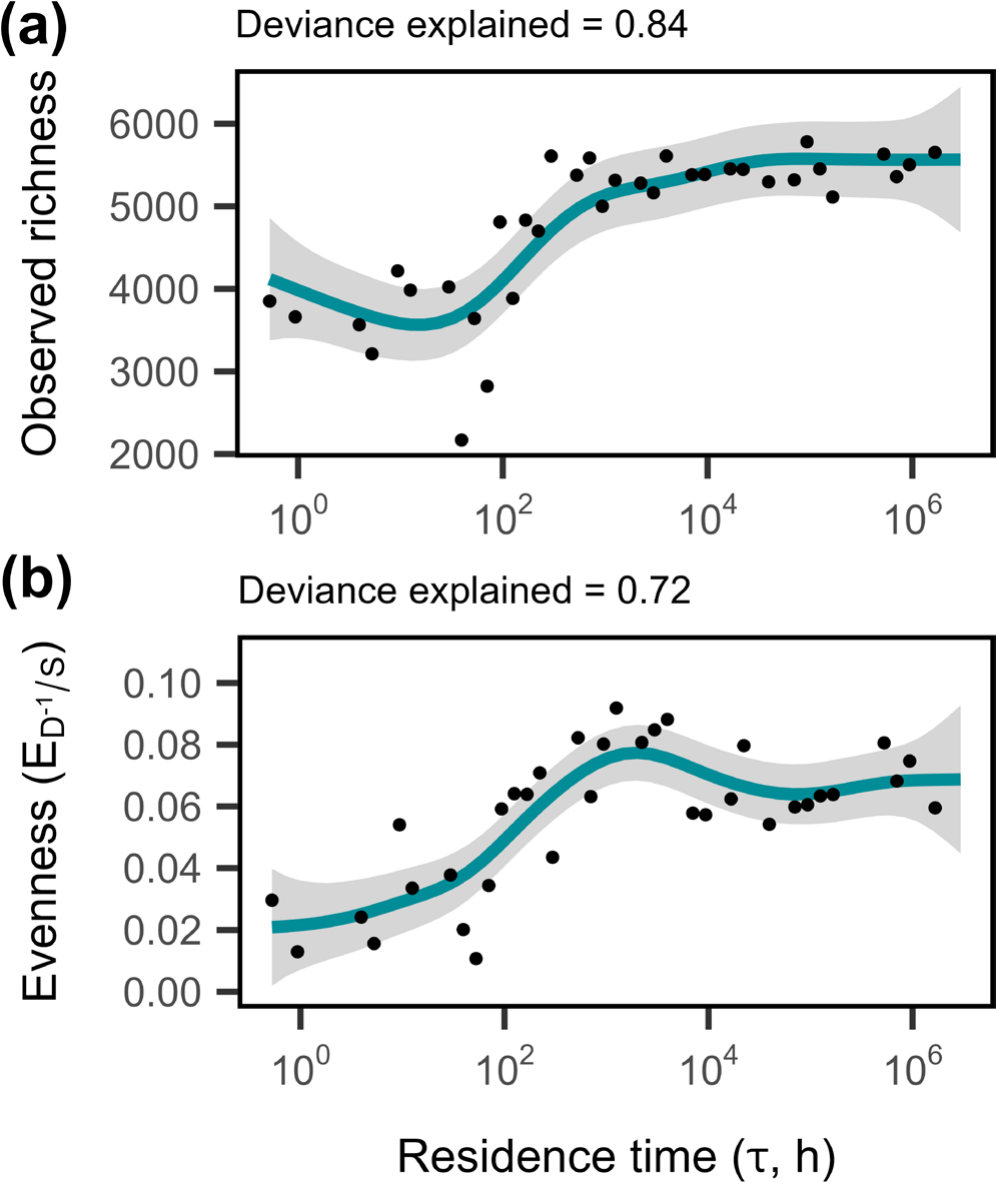
Microbial diversity increased with residence time. Generalised additive models (GAMs) describing the effect of residence time on (a) species richness (*S*), measured as the observed number of OTUs and (b) species evenness, calculated as Simpson's measure of evenness (*E* = *D* ^−1^/*S*; *D* ^−1^ is the inverse of Simpson's diversity). Lines and shading represent fits and 95% CIs of GAM regressions, respectively. The percentage of deviance explained by the model is reported above each plot (Table 1).

**FIGURE 4:**
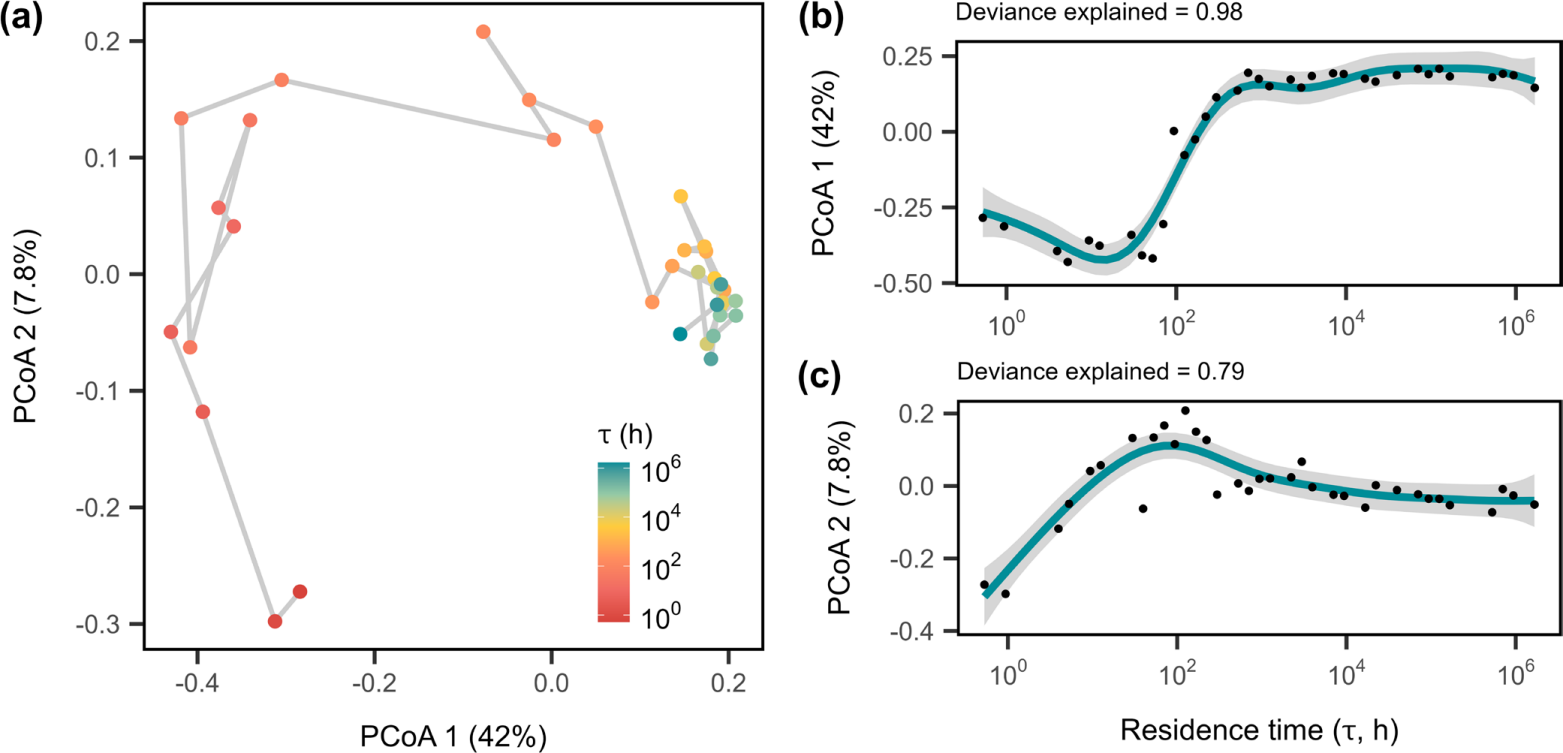
Community assembly along a residence time gradient. (a) Principal coordinate analysis (PCoA) plot showing how bacterial community composition responds to residence time after 20 days. Points are coloured by *τ* and are connected by a line indicating the relationship between the residence times. Generalised additive models (GAMs) show the effect of residence time on microbial community composition along the first two axes of the principal coordinate analysis, which account for (b) 42% and (c) 8% of the total variation, respectively. Lines and shading represent fits and 95% CIs of GAM regressions, respectively. The percentage of deviance explained by the model is reported above each plot (Table 1).

To characterise microbial diversity of the chemostats, we sequenced the 16S rRNA gene and recovered a total of 23,234 bacterial and 129 archaeal operational taxonomic units (OTUs). Chemostats were dominated by seven phyla common to freshwater ecosystems: *Proteobacteria* (14%–42%), *Planctomycetota* (11%–45%), *Acidobacteriota* (4%–24%), *Verrucomicrobiota* (5%–14%), *Bacteroidota* (3%–15%), *Actinobacteriota* (1%–11%) and *Chloroflexi* (1%–9%; Figure S4). Univariate measures of diversity were strongly affected by residence time. Taxon richness (*S*) increased non‐linearly with increasing residence time (Figure 3a, *F*_5.06_ = 18.2, *p* < 0.0001), ranging from 2000 to 6000 taxa, saturating at longer residence times. As residence time increased, evenness (*E*) also increased and plateaued at a value of 0.08 (Figure 3b, *F*_4.97_ = 11.5, *p* < 0.0001). Similar patterns of community richness and evenness were found when data were analysed as amplicon sequencing variants (ASVs), which have shown a higher sensitivity for strain detection (Supporting Information Methods; Figure S5; Table S1; Chiarello et al. 2022). Multivariate measures of diversity were also strongly affected by residence time. When visualised with PCoA, the first axis accounted for 42% of the variation in the dataset while the second axis accounted for 8% of the variation (Figure 4a). Residence time had a strong effect on the compositional trajectory in ordination space along the first principal coordinates axis (Figure 4a; PCoA 1, *t*_33_ = 8.3, *p* < 0.0001, *ρ* = 0.82). Additionally, community composition at the end of the experiment (Day 20) significantly diverged from that at the beginning of the experiment (Day 0; Figure S6a,b; PERMANOVA, *F*_1_ = 26.3, *p* = 0.001; see Supporting Information Results). When chemostat PCoA scores along the first two axes were plotted against residence time, they increased non‐linearly with increasing residence time (Table 1; Figure 4b, PCoA 1, *F*_7.88_ = 119.0, *p* < 0.0001; Figure 4c, PCoA 2, *F*_5.35_ = 15.7, *p* < 0.0001).

### 3.4 Residence Time Resulted in Niche Partitioning

**FIGURE 5:**
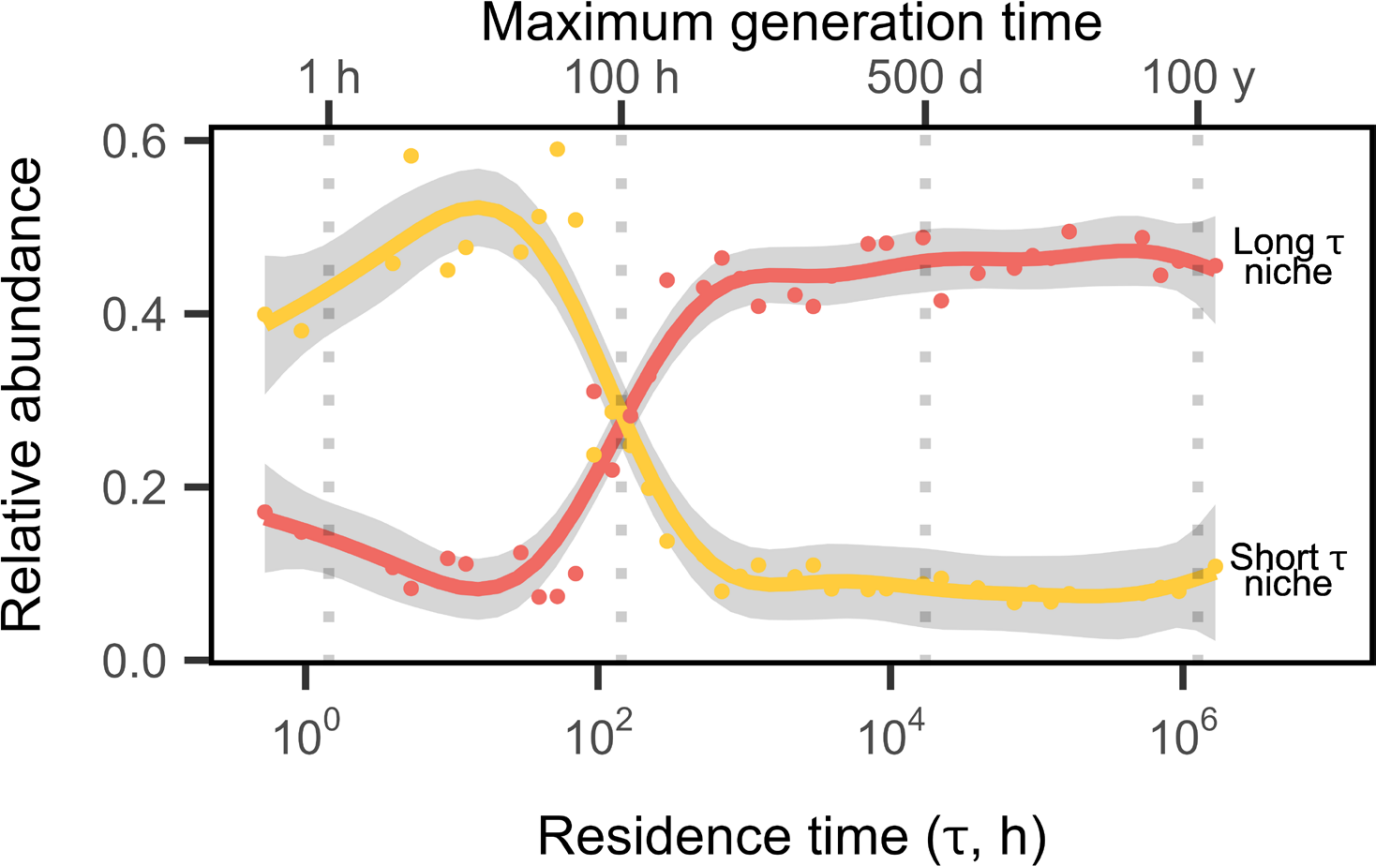
Niche partitioning along a residence time gradient. Total relative abundance of the two major niches identified through *k*‐means clustering is shown along the residence time axis. The long‐*τ* niche, shown in red is identified as ‘niche 1’ in the text and the short‐*τ* niche, shown in yellow, is identified as ‘niche 4’ in the text. Vertical grey dotted lines represent the maximum generation time organisms can have at a given residence time (top axis) which is equal to the dilution rate of a chemostat (1/*τ*) that would allow population sizes to remain at equilibrium. Generation times shorter than this maximum generation times would reduce the probability of washout. Lines and shading represent fits and 95% CIs of generalised additive model (GAM) regressions, respectively (Table 1).

Based on Pianka's overlap index, there was significantly less overlap in microbial taxa along the residence time gradient than expected under null models (Figure S7). Using *k*‐means clustering and minimisation of within groups sum of squares, taxa fell into five niches (Figure S8a,b, niche 1: *n* = 673, 30.5%; niche 2: *n* = 427, 19.4%; niche 3: *n* = 336, 15.3%; niche 4: *n* = 287, 13.0%; niche 5: *n* = 481, 21.8%). Two of these niches (niches 1 and 4) accounted for ~45% of the total relative abundance and were positioned at long and short residence times, respectively (Figure 5; Figure S8c). These two major niches were composed of 133 orders, 14 unique at short *τ*, 75 unique at long *τ* and 44 shared (Table S2). In contrast, the three minor niches (niches 2, 3 and 5) each accounted for only ~10% of the total relative abundance (see Supporting Information Results). Both major niches exhibited phylogenetic clustering, but the short‐*τ* niche had a stronger phylogenetic signal (*D* = 0.38, *P*_Random_ < 0.0001, Cohen's *d* = 19.14) than the long‐*τ* niche (*D* = 0.58, *P*_Random_ < 0.0001, Cohen's *d* = 15.36).

## 4 Discussion

We experimentally tested how residence time affects the assembly, diversity and function of a complex microbial community using an array of continuous flow reactors (i.e., chemostats). Patterns in bacterial abundance and productivity support the hypothesis that residence time structures communities through the opposing selective pressures of washout and resource limitation. These pressures altered community assembly and resource consumption in ways that led to niche partitioning along the residence time gradient. The phylogenetic distribution of taxa within the two major niches suggests that conserved traits may contribute to patterns of diversity and function that emerge when communities are exposed to variation in physical turnover.

### 4.1 Opposing Selective Forces: Washout vs. Resource Limitation

Theory predicts that washout is the primary factor constraining abundance and species richness at short residence times (Locey and Lennon 2019). Under such conditions, individuals are removed more quickly than they can be replaced via reproduction, which can lead to the local extinction of some species. In support of this, we observed lower bacterial abundances and lower taxon richness in chemostats with short residence times (Figures 1b and 3a). For the shortest residence time tested, a bacterium would have needed a growth rate of at least 1.9 h^−1^, which is equivalent to a doubling time of ~30 min. There are many clinically relevant strains of bacteria capable of replicating on this timescale, including *Escherichia coli* , *Klebsiella pneumoniae* and *Salmonella enterica* (Irwin et al. 2010; Liao et al. 2011; Silva et al. 2009). However, most environmental microorganisms do not divide this quickly. Based on genomically inferred doubling times, the vast majority of aquatic bacteria would not be able to persist in chemostats with such short residence times (Weissman et al. 2021). Therefore, while some microorganisms may avoid washout through fast growth rates, other bacteria may need to invest in traits such as adhesion and motility to persist in systems with short residence times.

At longer residence times, resource limitation becomes the dominant ecological force, constraining metabolism, abundance and species richness (Locey and Lennon 2019). In support of this, bacterial productivity and resource consumption declined in chemostats with increasingly long residence times (Figure 1c,d). As a result, per capita productivity rates were extremely low. Assuming an average cellular biomass of 26 fg C per cell (Troussellier et al. 1997), we estimate that doubling times of microorganisms at the longest *τ* may be as high as 135 days, which is multiple orders of magnitude lower than the residence times of the chemostats in which they were maintained. While the 20‐day experiment likely did not allow all the chemostats to reach steady state, our findings support the hypothesis that at long *τ*, starvation affected communities more strongly than washout. Under such conditions of low immigration and resource supply, microbes must conserve internal resources to meet their maintenance energy requirements and prevent starvation (Jones and Lennon 2010). When cells die, their biomass components can be recycled, potentially supporting the metabolism of surviving individuals. For example, in a comparative analysis of phylogenetically diverse soil bacteria, nearly all populations could survive in the absence of exogenous resource inputs for at least 1000 days (Shoemaker et al. 2021). If the experimental duration were extended, we would expect microbial abundance and richness to decline, reflecting selection for taxa able to persist under conditions of extreme resource limitation. This ability is presumably common given that most habitats on Earth have long residence times that lead to vanishingly low rates of resource supply (Ambrosetti et al. 2003; Basu and Pick 1997; Jönsson et al. 2004).

### 4.2 Niche Partitioning Along the Residence Time Gradient

Niche theory predicts that species must be sufficiently similar in their requirements to coexist in a given environment, yet have different enough niche requirements to avoid competitive exclusion (Levine and HilleRisLambers 2009). When communities are structured to minimise niche overlap, clusters of taxa can emerge that specialise in different sets of conditions (Dumbrell et al. 2010; Johnson et al. 2006). This clustering reflects niche partitioning, a process that allows diverse groups of organisms to survive and coexist along environmental gradients. In the context of residence time, communities may be structured by multiple covarying variables including resources and physical factors like sheer stress generated by flow rate. Given its multifaceted impact, residence time may create an abundance of complex niches as environmental gradients overlap and interact.

In our experiment, we found evidence for niche partitioning. There was less overlap in the relative abundances of taxa across the residence time gradient than expected compared to null models (Figure S7). Despite the wide range of residence times spanning many orders of magnitude, we identified only two major niches that contained half of all the individuals sequenced in our study. One niche comprised taxa specialising in short residence times, while the other comprised those specialising in long residence times. Some taxa found in the short‐*τ* niche belonged to the Pseudomonadota and Bacteroidota, which have been shown to increase at short *τ* (Vuono et al. 2015). Taxa belonging to Flavobacteriaceae were less abundant at short *τ* compared to long *τ*. However, the persistence of these taxa at short *τ*, despite the reduced number of species, suggests an ability to establish a population even under conditions that could otherwise lead to washout (Bulseco et al. 2024). In contrast, the long‐*τ* niche had a wider and asymmetric distribution. It was composed of more taxa than the short‐*τ* niche, but each had a lower relative abundance within the cluster (Figure 5; Table S2). The dominant taxa in this niche belonged to the Chloroflexota and Actinomycetota, consistent with observations made following a residence‐time manipulation in a full‐scale activated‐sludge wastewater treatment plant (Vuono et al. 2015). We also identified three minor niches that were less abundant and changed less dramatically along the residence time gradient, but which either overlapped with the major niches (niches 2 and 5) or occupied a unique transition point along the residence time axis (niche 3).

The distribution of taxa along the residence time gradient most likely reflects the underlying distribution of organismal traits (Locey and Lennon 2019). At short *τ*, fast growth rates may allow some taxa to sustain positive growth, as has been shown in dilution rate experiments where species growth rate was positively correlated with competitive ability at shorter residence times (Abreu et al. 2019). Additionally, a few traits, for example, adhesion and motility, could reduce washout for slower‐growing taxa (Ballyk et al. 1998). At long *τ*, there may be different ways for taxa to withstand resource limitation. For example, the number of enzyme classes present and the fractional share of rarer enzymes increased non‐linearly with residence time in wastewater microbial communities (Mansfeldt et al. 2019). This may help explain the two‐fold difference in the number of taxa in the two niches (Table S2). We also see evidence for differential resource use for communities composed of taxa mainly found in the short‐*τ* vs. long‐*τ* niche in multivariate space (Figure 2a,c), which cannot be entirely attributed to average resource use given its near‐linear decline with increased residence time (Figure 1d). Furthermore, differences in trait distributions along the residence time gradient might be reflected by phylogenetic patterns, assuming related taxa are more likely to share similar traits (Martiny et al. 2015). We found support for this, as taxa within a niche cluster were more phylogenetically related to one another than expected by chance. Additionally, there was a stronger phylogenetic signal for taxa associated with the short‐*τ* niche than those associated with long *τ*. This pattern suggests that phylogenetically clustered traits are likely more important to species persistence at short *τ* than at long *τ*.

To determine the mechanisms of niche partitioning, future studies should measure functional traits in systems with different residence times. This could be accomplished by isolating strains from different residence times and directly comparing functional traits such as growth rates, motility and resource use (Lennon et al. 2012). For example, we observed trends in biofilm production suggesting that attachment may contribute to persistence along a residence time gradient (Supporting Methods and Results; Figure S9). However, cultivation bias toward fast‐growing taxa may interfere with proper inference. Alternatively, shotgun metagenomic or metatranscriptomic sequencing could be used to perform a functional analysis, identifying relevant suites of genes and their relationship to residence time (Palanisamy et al. 2023).

### 4.3 Applying Residence Time Theory to Natural Systems

Residence time is associated with a wide range of biological phenomena in natural and managed ecosystems. In aquatic ecosystems, it is well known that rates of primary productivity and algal bloom emergence increase with residence time (Kim et al. 2022; Søballe and Kimmel 1987). Meanwhile, in host‐associated ecosystems, the severity of gastrointestinal disorders like Crohn's disease increases with residence time and is associated with reduced gut microbial diversity (Fischer et al. 2017; Ott et al. 2004). However, additional factors must be considered when using residence time theory to understand dynamics in more complicated settings. While *V/Q* serves as a reasonable approximation of residence time in an idealised system, in reality, there is a distribution of residence time. In a flowing system, some particles are removed faster than others due to chance and chaotic dynamics resulting from turbulence (Nauman 2008). Variation can also arise when individuals evade washout by finding physical refugia or flow‐path sinks, allowing slow‐growing organisms to establish themselves in an otherwise fast‐moving system (Drost et al. 2014). Residence time can also change over time, as seen in intermittent streams, seasonal hydrologic conditions or changes in gut retention time in sick patients (Blackman et al. 2021; Fischer et al. 2017). Switching between states of washout stress and resource stress has the potential to cause rapid changes in organismal abundance and species richness, preventing a stable and observable community response to residence time (Febria et al. 2012). Scaling up theory and findings from our small‐scale reactors represents a future challenge for applying the residence‐time framework to larger and more complex systems.

Despite these complications, residence time theory has potential applications in clinical, engineered and environmental ecosystems. For example, in the gastrointestinal (GI) tract, residence time can be modified with water intake, laxatives or dietary changes (Tropini et al. 2018). In cases where GI diseases select for harmful microorganisms and promote microbial overgrowth, manipulating residence time could provide an alternative intervention strategy that avoids antibiotics (Procházková et al. 2023). In bioproduct manufacturing, residence time is critical for production rate and yield, but reactors typically operate with only one or two species (Fogler 2006). Understanding the effects of residence times on more diverse assemblages opens up opportunities for enhanced control over production processes, especially in light of recent insight into how stochastic and deterministic ecological forces influence the stability of synthetic microbial consortia (Li and Müller 2023). Last, eutrophication management is of concern to public health due to the increasing prevalence of illnesses associated with harmful algal blooms (HABs) in freshwater and coastal ecosystems (Grattan et al. 2016). Some HAB traits, such as toxin production and nitrogen fixation, become more prevalent with increasing residence time (Zhao et al. 2022). If flow rates (*Q*) can be adjusted based on an understanding of residence time's influence on phytoplankton biomass and productivity, then it may be possible to mitigate algal blooms and their harmful effects (Cui et al. 2023). Future work should build on the whole‐community approach used here to investigate traits that drive niche partitioning, with the goal of developing a comprehensive understanding of how residence time affects communities in diverse ecosystems.

## Author Contributions

E.A.M. and J.T.L. designed the study; E.A.M. performed the experiments; E.A.M. and J.T.L. analysed data and wrote the paper.

### Peer Review

The peer review history for this article is available at https://www.webofscience.com/api/gateway/wos/peer‐review/10.1111/ele.70093.

## Supporting information


Data S1.

## Data Availability

Code to reproduce all analyses is available on GitHub: https://github.com/LennonLab/ResidenceTime_NichePartitioning and Zenodo: 10.5281/zenodo.14868323.
