# Supplementary material for: Residence Time Structures Microbial Communities Through Niche Partitioning
Source: Ecol Lett. 2025 Feb 26;28(2):e70093. doi: 10.1111/ele.70093 (PMC11862987; doi:10.1111/ele.70093)
Supplement: Supplementary file 1 — Data S1. [file ELE-28-0-s001.docx]

**SUPPLEMENTAL INFORMATION – METHODS**

***Residence time in University Lake —*** We estimated the hydrological inflow (*Q*) to University Lake based on the difference between whole-basin area (720,017 m^2^) precipitation (1.264 m/y) and evapotranspiration rates (0.649 m/y) assuming negligible net groundwater exchange. Based on this, we estimated a flow rate of 442,810 m^3^/y. Assuming a lake volume (*V*) of 150,000 m^3^ (Wisnoski *et al.* 2020), University Lake has a residence time of approximately 124 d.

***Experimental design —*** We ran chemostats in four experimental blocks (coded as “Set” in analyses) of ~12 chemostats each. Residence times of the chemostats in each experimental block spanned the entire residence time gradient (~30 min to 330 y) to minimize block-specific effects on one portion of the gradient. For residence times < 41 h (1.7 d), we used peristaltic pumps to turnover the chemostats. For residence times > 41 h (1.7 d), we manually added and removed volume from the chemostats once per day with a pipette.

***Flow cytometry*** ***—*** Redox sensor green (RSG) green fluorescence events were collected on a logarithmic scale and set to a gain of 453 at a speed of 14 µL/min. We collected a total of 10,000 events with a forward scatter height (FSC-H) larger than 300. We removed non-singleton events by gating on a side scatter area vs. side scatter height (SSC-H vs. SSC-A) plot (Fig. S2A). Next, we gated for live cells on an RSG vs. count plot to remove cytometer noise seen in cell-free phosphate buffered saline (PBS) samples while using the location of the unstained sample on the RSG axis to avoid removing live cells (Fig. S2B).

***DNA extraction and PCR conditions*** ***—*** We extracted genomic DNA from samples taken at the beginning (Day 0) and end (Day 20) of the chemostats experiment using the DNeasy UltraClean Microbial Kit (Qiagen, Germantown, MA, USA). For DNA extraction, after initial centrifugation of the samples, we incubated the bacterial resuspension in PowerBead Solution with lysozyme (50 µL 50 mg/mL) and Proteinase K (10 µL 20 mg/mL) for 1 h at 37 °C. We then proceeded according to the UltraClean kit instructions. DNA was eluted in 30 µL EB Buffer and stored at -20 °C. We amplified the V4 hypervariable region of the 16S rRNA gene using barcoded primers (515F and 806R) designed for the Illumina MiSeq platform (Caporaso *et al.* 2012). We used Phusion High Fidelity DNA Polymerase (New England BioLabs, Ipswich, MA, USA) to run 25 µL reactions with the 5X Phusion HF buffer, 0.75 µL DMSO, and 0.5 µL template DNA. The conditions for the PCR reaction were 98 °C for 30 s, 30 cycles of 98 °C for 10 s, 68 °C for 30 s, and 72 °C for 15 s, followed by a final extension at 72 °C for 10 min.

***Sequencing* *—*** We purified the sequence libraries using the AMPure Purification Kit (Beckman Coulter, Brea, CA, USA) and quantified the libraries with the Quant-it PicoGreen dsDNA kit (Invitrogen, Waltham, MA, USA). We then pooled the libraries to equal molar ratios (final concentration of 10 ng/library) and sequenced them using Illumina MiSeq 250x250 paired end reads (Reagents v2) at the Indiana University Center for Genomics and Bioinformatics.

***Diversity and assembly —*** We processed raw 16S rRNA sequences to determine taxonomic composition of each chemostat along the residence time gradient. We assembled the paired-end raw 16S rRNA sequence reads into contigs, quality-trimmed, and aligned them to the Silva Database (version 138; Quast et al. 2013). We detected and removed chimeric sequences using the VSEARCH algorithm (Rognes *et al.* 2016). We then split the sequences based on RDP taxonomy (Cole *et al.* 2009) then binned them into operational taxonomic units (OTUs) based on 97% sequence similarity using the OptiClust method (Westcott & Schloss 2017). These initial sequence processing steps were completed using the software package mothur (Schloss 2020; Schloss *et al.* 2009). We also calculated phylogenetic distance between OTUs and generated a phylogenetic tree using the ‘dist.seqs’ method and Clearcut which implements a relaxed neighbor joining algorithm (Evans *et al.* 2006), respectively, both implemented in mothur. We removed OTUs with less than two reads across all samples.

***Amplicon sequence variant (ASV) diversity and assembly —*** To determine the robustness of α-diversity patterns with residence time to clustering vs. denoising methods, we also used DADA2 (version 1.16.0, Callahan et al. 2016) to generate ASVs. We filtered and quality-trimmed the raw 16S rDNA sequence reads and then merged the paired reads. We then removed chimeras and assigned taxonomy to the sequence variants using the Silva Database (version 138). We calculated observed ASV richness (*S*) and evenness (*E*) after rarefication (*n* = 21,515).

***Community composition —*** To test for differences in community composition between the beginning (Day 0) and end (Day 20) of the experiment, we used a PERMANOVA on Bray-Curtis distances using the ‘adonis2’ function from the ‘vegan’ package (version 2.6-4; Oksanen et al. 2012) and calculated pairwise Bray-Curtis distances for paired Day 0 and Day 20 samples in each chemostat. We also compared taxonomic makeup at the beginning and end of the experiment, categorizing rare taxa as those making up the bottom 1% of total relative abundance and common taxa as those making up the bottom 99% of total relative abundance. We then calculated the percentage of common taxa present on Day 20 that were categorized as rare on Day 0.

***Biofilm production*** ***—*** We measured biofilm production using a crystal violet assay (O’Toole *et al.* 1999). We added 100 µL of autoclaved lake-water medium into 96-well plates and inoculated replicate wells (*n* = 8) with 5 µL of sample from the end of the experiment (Day 20) after vortexing the chemostat to homogenize planktonic and attached cells. We incubated the plates at 25 °C for 96 h before adding 25 µL of 1% crystal violet to each well. After 20 min of incubation, we removed all liquid from the wells, rinsed the plates, and let them dry inverted on a paper towel overnight. We added 250 µL of 95% EtOH to all wells for 1 h before measuring the absorbance of the wells at 550 nm using a BioTek Synergy H1 (Agilent, Santa Clara, CA, USA). We estimated per cell biofilm production as:

$$\begin{aligned} Per cell biofilm production= \frac{Total biofilm produced}{N\times0.005}\#\left( S1 \right) \end{aligned}$$

where *N* is the microbial abundance in cells/mL, as 5 µL was used to inoculate each well.

**SUPPLEMENTAL INFORMATION – RESULTS**

***Characterization of individual carbon source use —*** The relationship between use of individual carbon sources and residence time can be grouped into near linear decline, exponential decline, and sigmoidal decline with increased residence times. Sigmoidal decreasing carbon sources were Tween 40, Tween 80, Pyruvic Acid Methyl Ester, L-Serine, L-Asparagine, and D-Galactonic Acid γ-Lactone. Exponentially decreasing carbon sources were Glucose-1-Phosphate and D-Xylose. Near linearly decreasing carbon sources were Putrescine, L-Argine, Glycyl-L-Glutamic Acid, D-Glucosaminic Acid, D-Galacturonic Acid, 4-Hydroxy Benzoic Acid and γ-Hydroxybutyric Acid. L-Threonine was the only carbon source where use increased significantly along the residence time gradient.

***Community composition*** ***—*** Average pairwise distances (Bray-Curtis) between community composition at the beginning of the experiment (Day 0) and the end of the experiment (Day 20) ranged from 0.74 at short τ to 0.49 at long τ (Fig. S6B). At short τ, ~38% (*n* = 1,428) of the taxa found to be common (making up the top 99% of taxa; *n* = 3,447) at the end of the experiment (Day 20) were rare (making up the bottom 1% of taxa; *n* = 26992) at the beginning of the experiment (Day 0). At long τ, ~51% (*n* = 2,614) of the taxa common at the end of the experiment (*n* = 5,099) were rare at the beginning.

***Characterization of minor niches —*** Despite each making up only ~10% of total relative abundance, the three minor niches show distinct trajectories along the residence time gradient. Niche 3 shows increased abundances at intermediate residence times (τ = 10^2^), perhaps occupying the transition between the long-residence-time niche (niche 1) and the short-residence-time niche (niche 4). Niche 2 shows an increased abundance at long residence times, reaching a maximum relative abundance at approximately τ = 10^4^. Niche 5 also appears to increase in relative abundance as residence times increase, peaking at approximately τ = 10^6^.

***Biofilm production*** ***—*** Total community biofilm production increased with residence time (Table S1, Fig. S9A, *F_3.29_* = 6.1, *P* = 0.0005). When expressed on a per cell basis, biofilm production was highest at short residence times (Table S1, Fig. S9B, *F_2.73_* = 17.7, *P* < 0.0001). However, owing to batch effects, the experimental block was a stronger predictor of biofilm production than residence time (Table S1, Total community biofilm, *F_2.89_* = 30.4, *P* = 0.0005; Per cell biofilm, *F_1.92_* = 40.3, *P* < 0.0001).

**SUPPLEMENTAL INFORMATION – REFERENCES**

Callahan, B.J., McMurdie, P.J., Rosen, M.J., Han, A.W., Johnson, A.J.A. & Holmes, S.P. (2016). DADA2: High-resolution sample inference from Illumina amplicon data. *Nat. Methods*, 13, 581–583.

Caporaso, J.G., Lauber, C.L., Walters, W.A., Berg-Lyons, D., Huntley, J., Fierer, N., *et al.* (2012). Ultra-high-throughput microbial community analysis on the Illumina HiSeq and MiSeq platforms. *ISME J.*, 6, 1621–1624.

Cole, J.R., Wang, Q., Cardenas, E., Fish, J., Chai, B., Farris, R.J., *et al.* (2009). The Ribosomal Database Project: improved alignments and new tools for rRNA analysis. *Nucleic Acids Res.*, 37, D141–D145.

Evans, J., Sheneman, L. & Foster, J. (2006). Relaxed neighbor joining: A fast distance-based phylogenetic tree construction method. *J. Mol. Evol.*, 62, 785–792.

Oksanen, J., Blanchet, F.G., Friendly, M., Kindt, R., Legendre, P., McGlinn, D., *et al.* (2012). vegan: Community Ecology Package.

O’Toole, G.A., Pratt, L.A., Watnick, P.I., Newman, D.K., Weaver, V.B. & Kolter, R. (1999). Genetic approaches to study of biofilms. *Methods Enzymol.*, 310, 91–109.

Quast, C., Pruesse, E., Yilmaz, P., Gerken, J., Schweer, T., Yarza, P., *et al.* (2013). The SILVA ribosomal RNA gene database project: improved data processing and web-based tools. *Nucleic Acids Res.*, 41, D590–D596.

Rognes, T., Flouri, T., Nichols, B., Quince, C. & Mahé, F. (2016). VSEARCH: a versatile open source tool for metagenomics. *PeerJ*, 4, e2584.

Schloss, P.D. (2020). Reintroducing mothur: 10 Years Later. *Appl. Environ. Microbiol.*, 86, e02343-19.

Schloss, P.D., Westcott, S.L., Ryabin, T., Hall, J.R., Hartmann, M., Hollister, E.B., *et al.* (2009). Introducing mothur: Open-Source, Platform-Independent, Community-Supported Software for Describing and Comparing Microbial Communities. *Appl. Environ. Microbiol.*, 75, 7537–7541.

Westcott, S.L. & Schloss, P.D. (2017). OptiClust, an improved method for assigning amplicon-based sequence data to operational taxonomic units. *mSphere*, 2, e00073-17.

Wisnoski, N.I., Muscarella, M.E., Larsen, M.L., Peralta, A.L. & Lennon, J.T. (2020). Metabolic insight into bacterial community assembly across ecosystem boundaries. *Ecology*, 101, e02968.

**SUPPLEMENTAL INFORMATION – TABLES**

| **Fit Model** | **Deviance Explained** | **Model**  ***P-*value** | **Term** | **Edf** | ***F-*statistic** | ***P-*value** | **REML** |
| --- | --- | --- | --- | --- | --- | --- | --- |
| Total biofilm production | 0.74 | 0.0002 | s(τ) | 3.29 | 6.14 | 0.0005 | -71.3 |
|  |  |  | s(Set, bs = re) | 2.89 | 30.37 | < 0.0001 |  |
| Per cell biofilm production | 0.82 | < 0.0001 | s(τ) | 2.73 | 17.66 | < 0.0001 | 9.98 |
|  |  |  | s(Set, bs = re) | 1.92 | 40.26 | < 0.0001 |  |
| ASV richness | 0.74 | < 0.0001 | s(τ) | 5.52 | 10.96 | < 0.0001 | 235.2 |
| ASV evenness | 0.83 | 0.0053 | s(τ) | 5.55 | 13.59 | < 0.0001 | -41.3 |
|  |  |  | s(Set, bs = re) | 2.39 | 3.56 | 0.0105 |  |
| Niche 2 | 0.62 | < 0.0001 | s(τ) | 4.11 | 9.06 | < 0.0001 | -79.4 |
| Niche 3 | 0.63 | < 0.0001 | s(τ) | 3.35 | 11.80 | < 0.0001 | -59.9 |
| Niche 5 | 0.95 | < 0.0001 | s(τ) | 7.04 | 61.39 | < 0.0001 | -46.4 |
| D-Galactonic Acid γ-Lactone | 0.38 | < 0.0001 | s(τ) | 2.85 | 7.26 | 0.0003 | -1.5 |
| D-Galacturonic Acid | 0.12 | < 0.0001 | s(τ) | 1.00 | 6.29 | 0.0156 | 6.5 |
| D-Glucosaminic Acid | 0.28 | < 0.0001 | s(τ) | 1.55 | 9.87 | 0.0007 | -34.0 |
| D-Xylose | 0.40 | < 0.0001 | s(τ) | 2.02 | 11.57 | < 0.0001 | -38.6 |
| γ-Hydroxybutyric Acid | 0.20 | < 0.0001 | s(τ) | 1.00 | 11.48 | 0.0014 | 18.6 |
| Glucose-1-Phosphate | 0.32 | < 0.0001 | s(τ) | 2.92 | 4.95 | 0.0028 | -11.4 |
| Glycyl-L-Glutamic Acid | 0.21 | < 0.0001 | s(τ) | 2.48 | 3.42 | 0.0231 | -62.1 |
| L-Arginine | 0.31 | < 0.0001 | s(τ) | 1.47 | 10.35 | 0.0003 | 6.5 |
| L-Asparagine | 0.54 | < 0.0001 | s(τ) | 3.79 | 10.44 | < 0.0001 | 13.0 |
| L-Serine | 0.41 | < 0.0001 | s(τ) | 4.04 | 5.19 | 0.0008 | 7.2 |
| L-Threonine | 0.45 | < 0.0001 | s(τ) | 4.86 | 5.17 | 0.0004 | -24.8 |
| Putrescine | 0.37 | < 0.0001 | s(τ) | 1.00 | 27.16 | < 0.0001 | 0.7 |
| Pyruvic Acid Methyl Ester | 0.50 | < 0.0001 | s(τ) | 3.88 | 8.41 | < 0.0001 | -3.2 |
| Tween 40 | 0.77 | < 0.0001 | s(τ) | 4.86 | 23.46 | < 0.0001 | -26.5 |
| Tween 80 | 0.64 | < 0.0001 | s(τ) | 4.27 | 13.74 | < 0.0001 | -10.7 |

**Table S1. Output from generalized additive models for supplemental figures.** Response variables for each generalized additive model (Fit model) are shown with deviance explained and *P*-value for the total model (Model *P*-Value). Each smoothed term (Term) has an effective degree of freedom (edf) and *F*-value (*F*-statistic). Significance of each smoothed term (Term) was determined from term *P*-values (*P*-value) (α = 0.05). Restricted maximum likelihood values (REML) for each model are shown.

| **Niche** | **Phylum** | **Order** | **# OTUs** |
| --- | --- | --- | --- |
| Short τ  (*n* = 287) | Bacteroidota | Flavobacteriales | 8 |
|  | Pseudomonadota | Acetobacterales | 9 |
| Long τ  (*n* = 673) | Actinomycetota | Gaiellales | 28 |
|  |  | IMCC26256 | 10 |
|  |  | MC-A2-108 | 9 |
|  | Chloroflexota | Anaerolineales | 18 |
|  |  | JG30-KF-CM66 | 6 |
|  |  | KDA-96 | 21 |
|  | Latescibacterota | Latescibacterota | 6 |
|  | Methylomirabilota | Rokubacteriales | 8 |

**Table S2. Some taxonomic orders are specialists for short τ or long τ.** Some orders are found exclusively in either the short-τ or long-τ niche, containing a notable number of representative OTUs (> 5). These orders are grouped by phylum within each niche and the number of OTUs in the taxonomic cluster is reported. The total number of OTUs in the niche are also reported (Short-τ niche – *n* = 287; Long-τ niche – *n* = 673).

**SUPPLEMENTAL INFORMATION – FIGURE CAPTIONS**

**Fig. S1. Community composition and resource use do not reflect mode of flow-rate manipulation.** At high flow (> 1.2 mL/h), chemostats were supplied with media using peristaltic pumps, while at lower flow (< 1.2 mL/h) peristaltic pumps could not be used to accurately control the media supply. Thus, we manually supplied those chemostats with media using a pipette. Our data indicate that residence-time-based changes observed in both community composition and resource use did not correspond with any artifactual break-points in reported patterns. Principal coordinate analysis (PCoA) plots show **(A)** community composition and **(B)** resource use responses to residence time after 20 d with black circles representing manual pipetting and blue circles representing peristaltic pumps.

**Fig. S2. Flow cytometry gating for microbial abundance. (A)** Cells were gated on a side scatter height vs. side scatter area (SSC-H vs. SSC-A) plot for single-celled organisms to remove aggregates and a portion of baseline cytometer noise from the sample. **(B)** On an RedoxSensor Green (RSG) fluorescence count plot, cells were gated for RSG activity. This gate was positioned to remove machine noise captured with unstained phosphate buffered saline (PBS) samples while avoiding removal of cells that were found in the unstained sample, meaning that live and active cells, regardless of activity level, were captured with this gate.

**Fig. S3. Use of individual carbon sources mainly decreases with increased residence time.** Individual carbon source use was measured with a colorimetric use assay (BioLog EcoPlate) as color formation measured at OD (590 nm) at 48-h. Only resources with significant relationships with residence time after Benjamini-Hochberg multiple testing corrections are shown. Lines and shading represent fits and 95% CIs of GAM regressions (Table S1).

**Fig. S4. Phylum level changes across the residence time gradient.** Relative abundance data was aggregated by phyla (*n* = 60) and fit with local polynomial regression (LOESS). Seven phyla, *Proteobacteria* (14-42%), *Planctomycetota* (11-45%), *Acidobacteriota* (4-24%), *Verrucomicrobiota* (5-14%), *Bacteroidota* (3-15%), *Actinobacteriota* (1-11%), and *Chloroflexi* (1-9%), dominated the microbial communities regardless of residence time.

**Fig. S5. Amplicon sequence variant richness and evenness reflect operational taxonomic unit richness and evenness.** Amplicon sequence variants (ASVs) are an alternative to operational taxonomic units (OTUs) and have been shown to have higher sensitivity for strain detection. **(A)** ASV richness, calculated as observed ASVs, increases along the residence time gradient, following the same pattern as OTU richness (Fig. 3A) **(B)** ASV evenness, calculated as Simpson’s measure of evenness (*E* = *D*^-1^/*S*; D^-1^ is the inverse of Simpson’s diversity), also increases with residence time in a similar manner to OTU evenness (Fig. 3B). Lines and shading represent fits and 95% CIs of GAM regressions and percentage of deviance explained by the model is reported above each plot (Table S1).

**Fig. S6. Community composition over time.** **(A)** Principal coordinate analysis (PCoA) plot showing how bacterial community composition changed from the beginning (Day 0 = circles) to the end (Day 20 = triangles) of the chemostats experiment. Points are colored by τ and the Day 20 samples are connected by a line indicating a relationship between the residence times. The first two axes of the PCoA account for 28% and 22% of total variation, respectively. **(B)** Pairwise Bray-Curtis distance between Day 0 and Day 20 samples show significant turnover across the residence time gradient, ranging from 0.74 at short τ to 0.49 at long τ.

**Fig. S7. Observed niche overlap was lower than expected from null models.** The first null model (Null model #1) pulled all chemostat-by-OTU relative abundances from a uniform distribution [0,1]. This null model had a mean Pianka’s niche overlap of 0.76 with a Cohen’s *d* value of 175.19. The second null model (Null model #2) maintained chemostat-by-OTU relative abundances that were zero, pulling the rest of the abundances from a uniform distribution [0,1]. This null model distribution had a mean Pianka’s niche overlap of 0.64 with a Cohen’s *d* value of 45.44. Both null model distributions showed higher niche overlap than what was observed across all chemostats and OTUs, which is a Pianka’s niche overlap of 0.61.

**Fig. S8. Five niches were identified across the residence time gradient using *k*-means clustering. (A)** Within group sum of squares of clusters fit with a LOESS regression indicated five niches as the optimal number of clusters. **(B)** Total relative abundance of all five niches identified through *k*-means clustering is shown along the residence time axis. Lines and shading represent fits and 95% CIs of GAM regressions (Table 1 & S1). **(C)** Relative abundance patterns for individual OTUs within the two major niches follow similar trajectories to the total relative abundance of all OTUs within each niche.

**Fig. S9. Biofilm production across the residence time gradient.** **(A)** Total biofilm production increased with residence time suggesting that cell aggregation and attachment may be promoted by the effects of resource limitation that are associated with reduced flow rates. **(B)** However, when standardized on a per cell basis, biofilm production decreased with residence time, suggesting attachment may be a strategy that prevents washout. Despite these overall patterns, we detected a significant effect of experimental block. Given the variation among chemostats using the crystal violet assay, future studies should explore relationships between residence time and biofilm production using other methods, including shotgun metagenomics. Points are colored by experimental block. Lines and shading represent fits and 95% CIs of GAM regressions and percentage of deviance explained by the model is reported above each plot (Table S1).

**Fig. S1.**


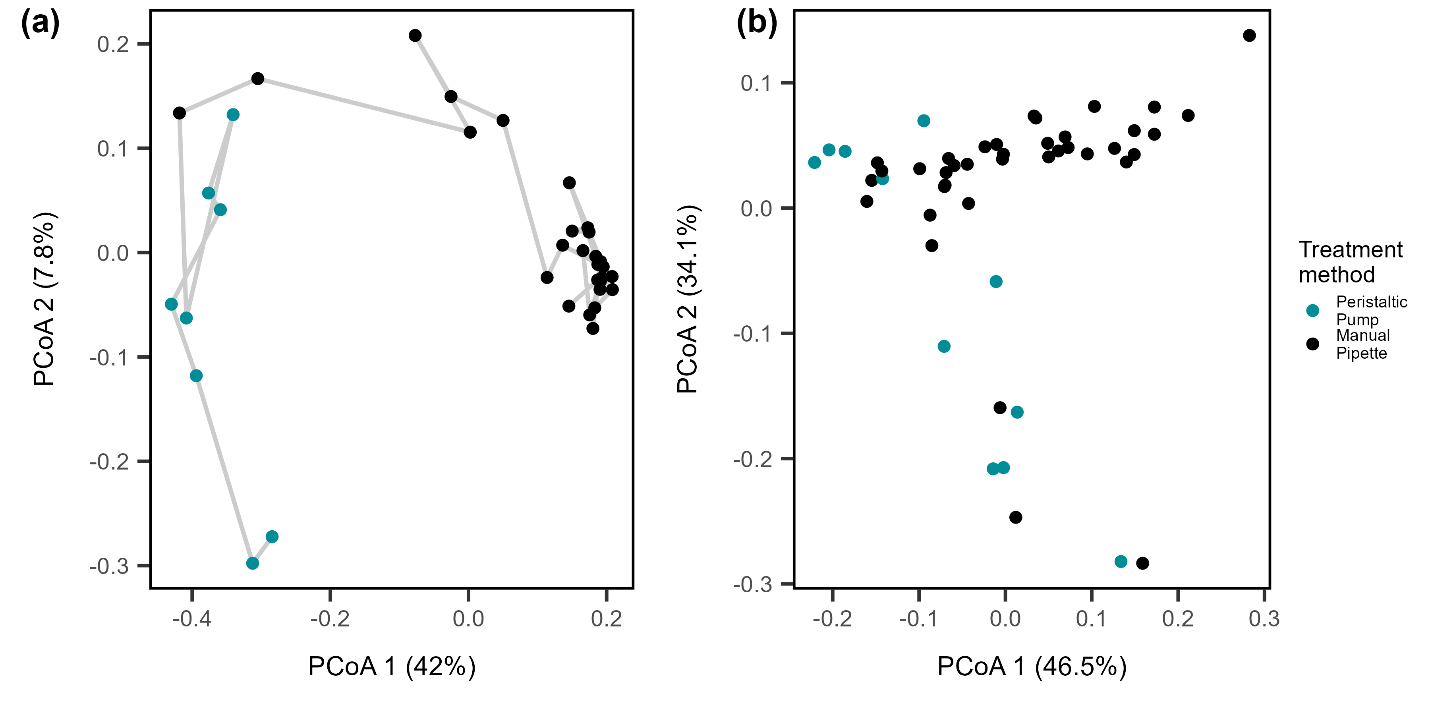


**Fig. S2.
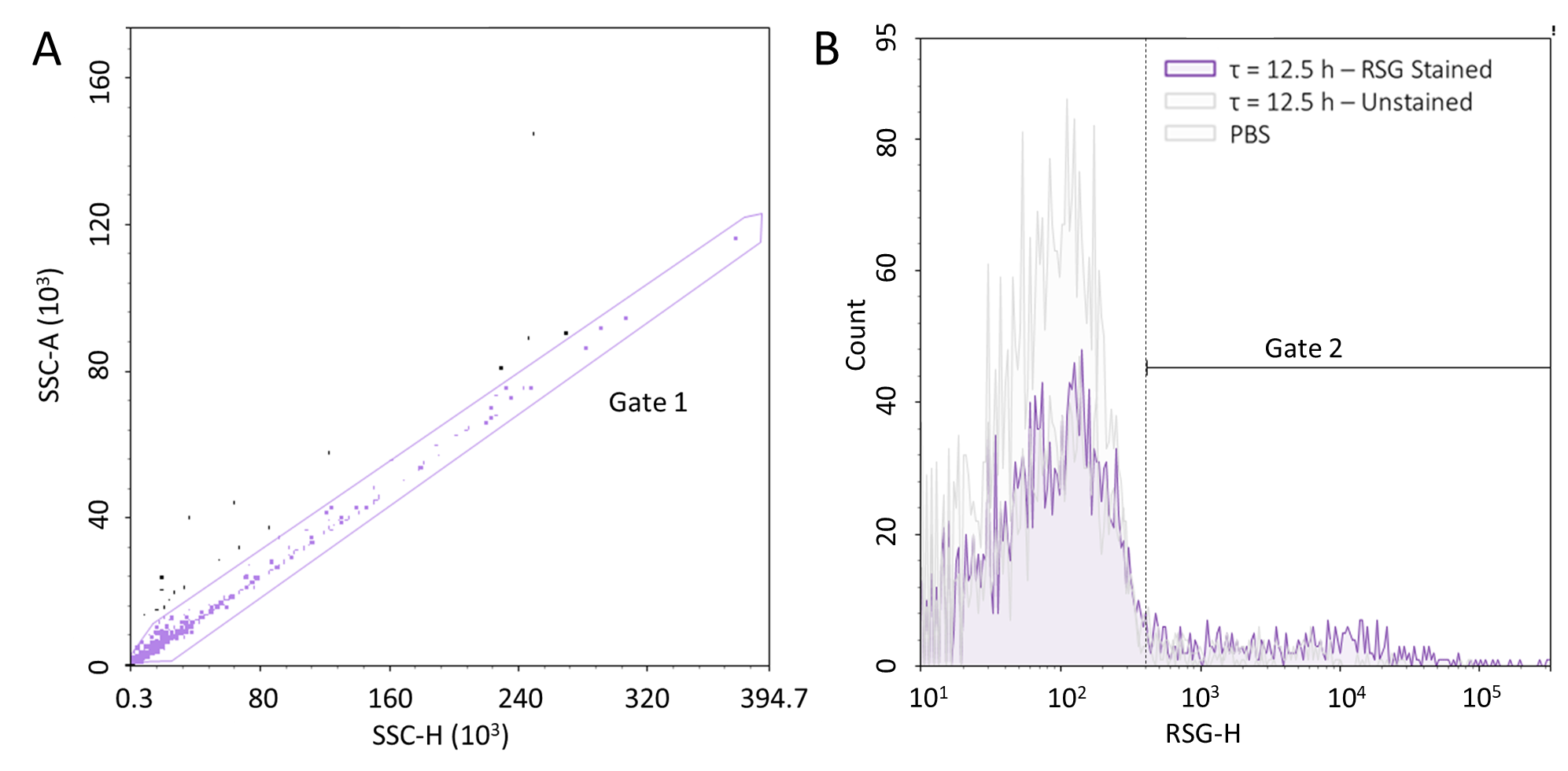
**

**Fig. S3.**

**
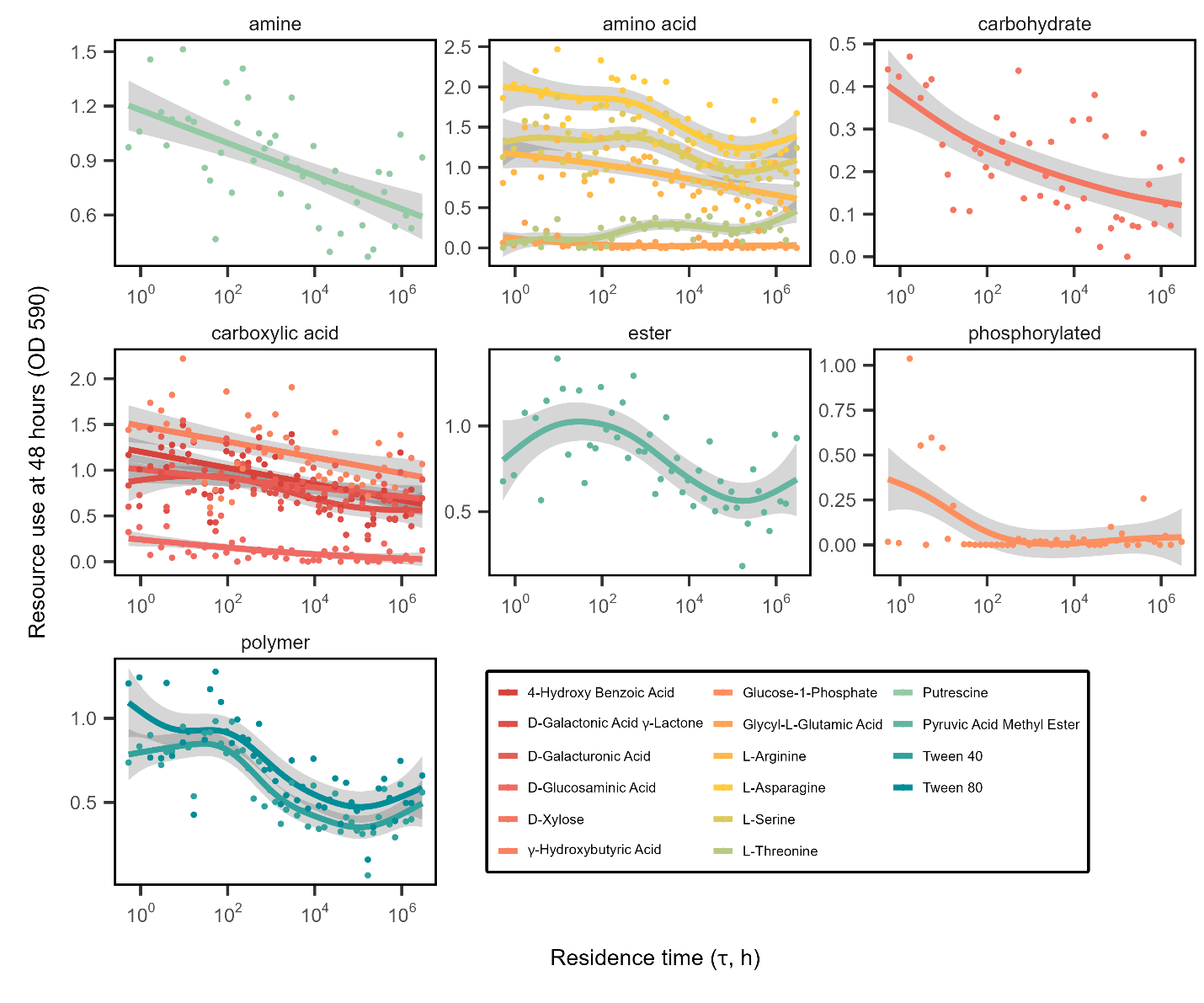
**

**Fig. S4.**


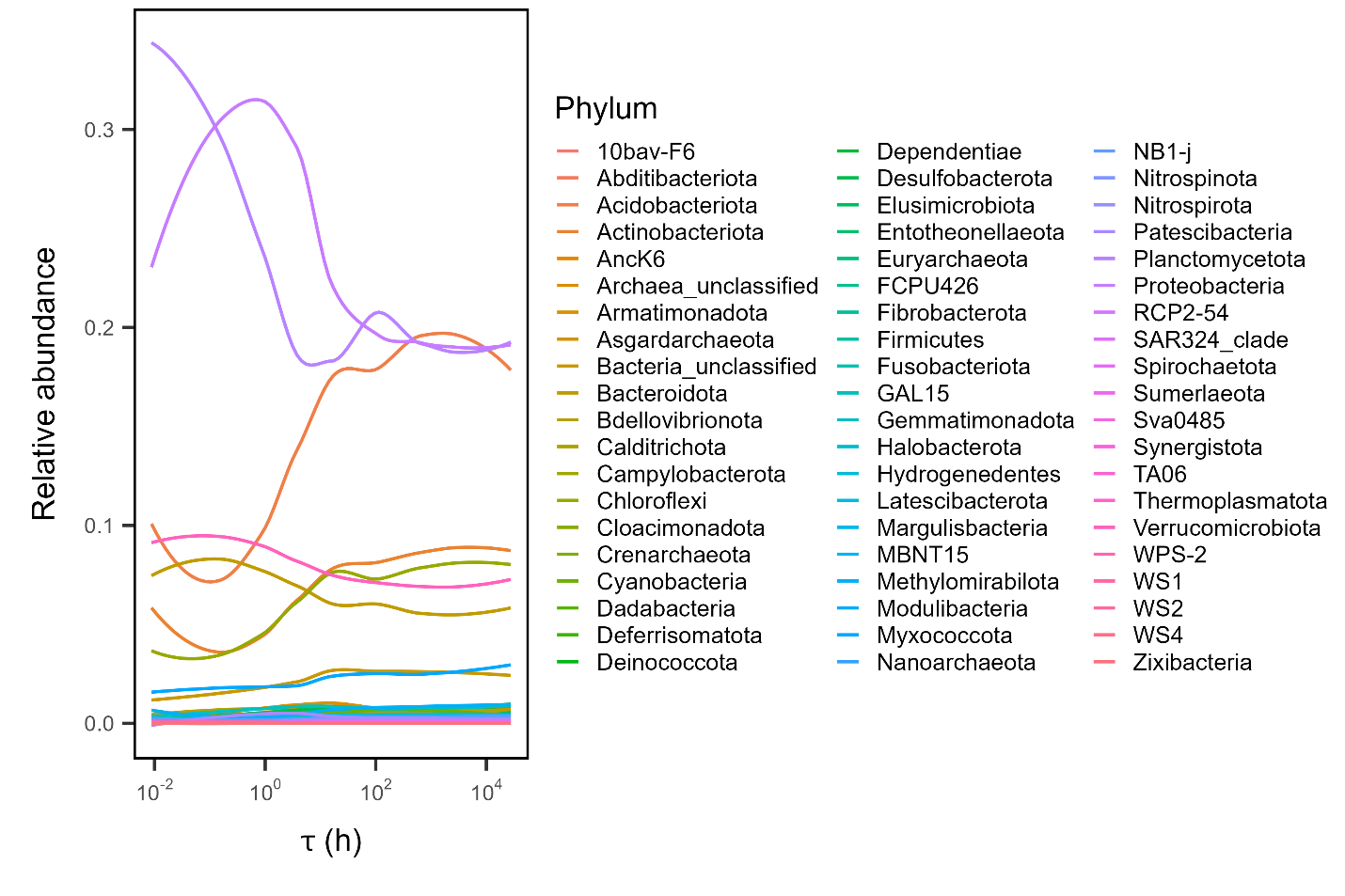


**Fig. S5.**


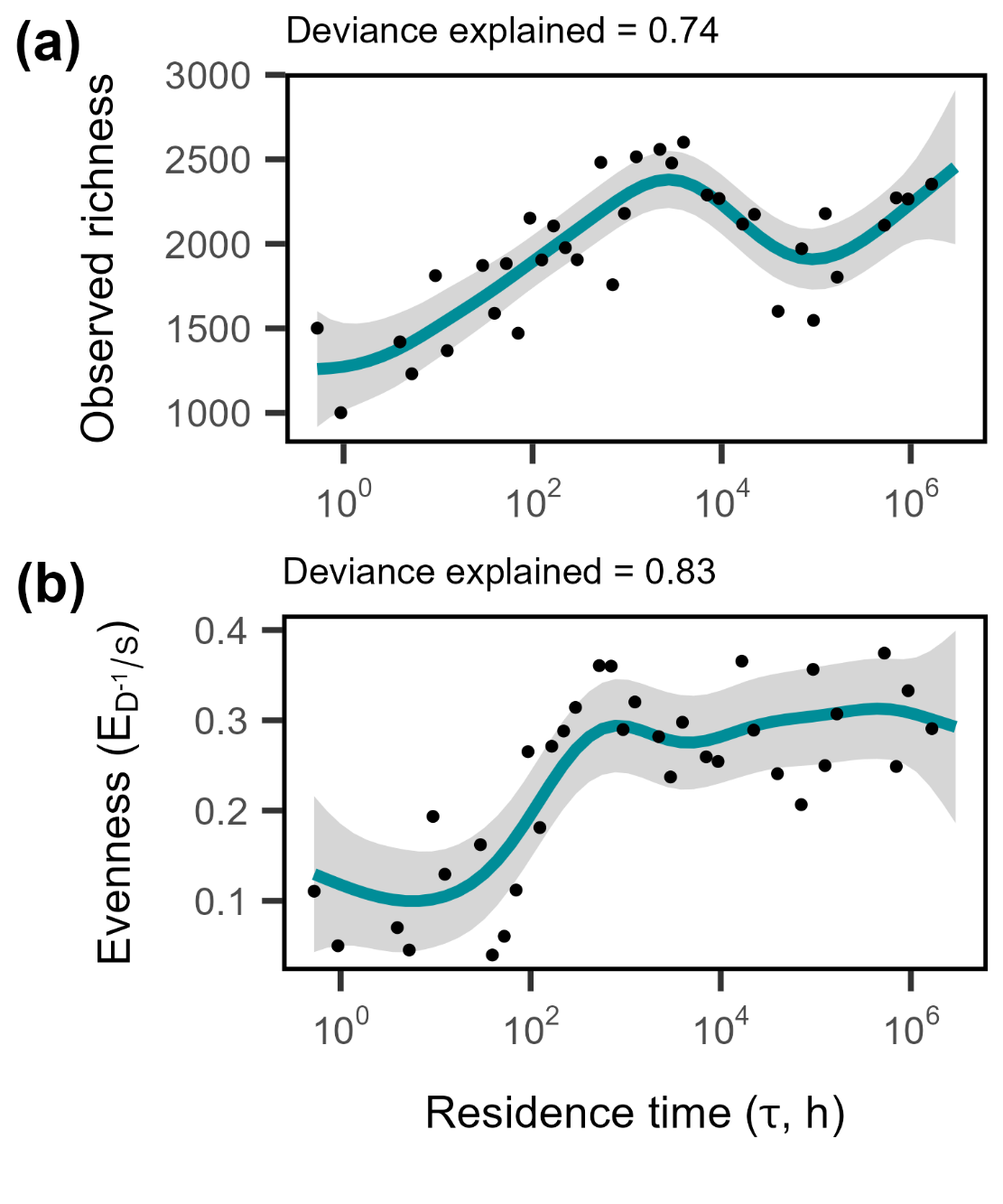


**Fig. S6.**

**
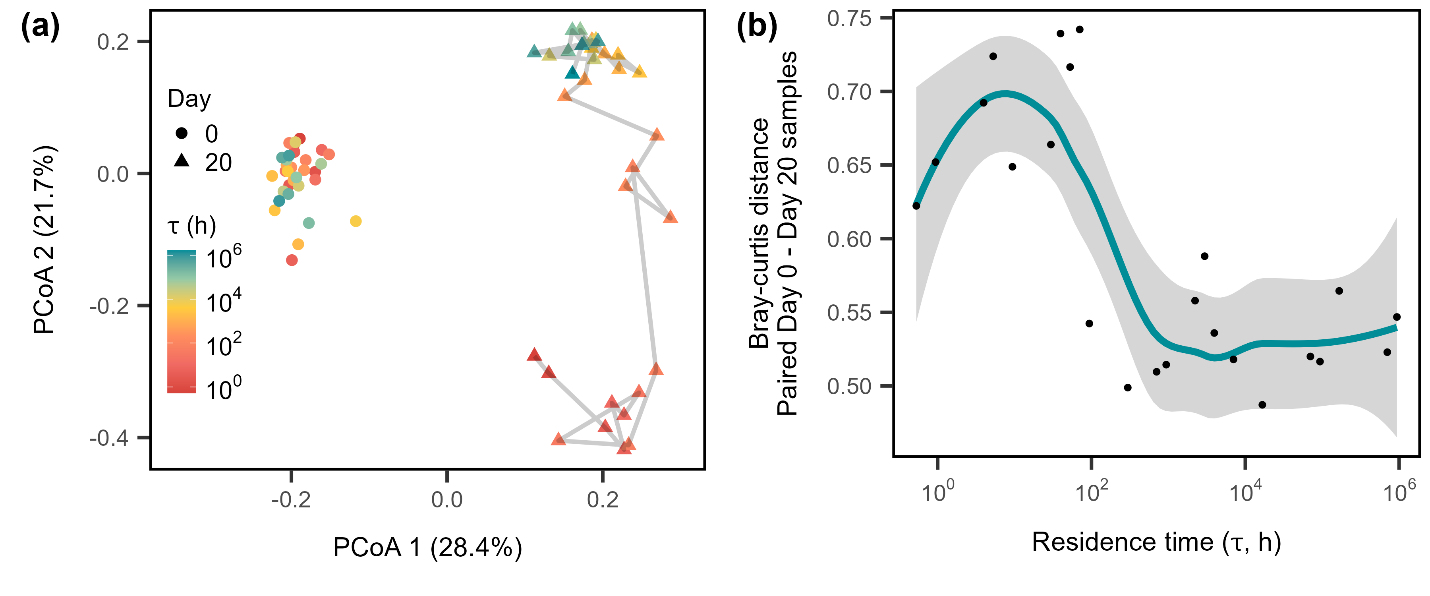
**

**Fig. S7.**


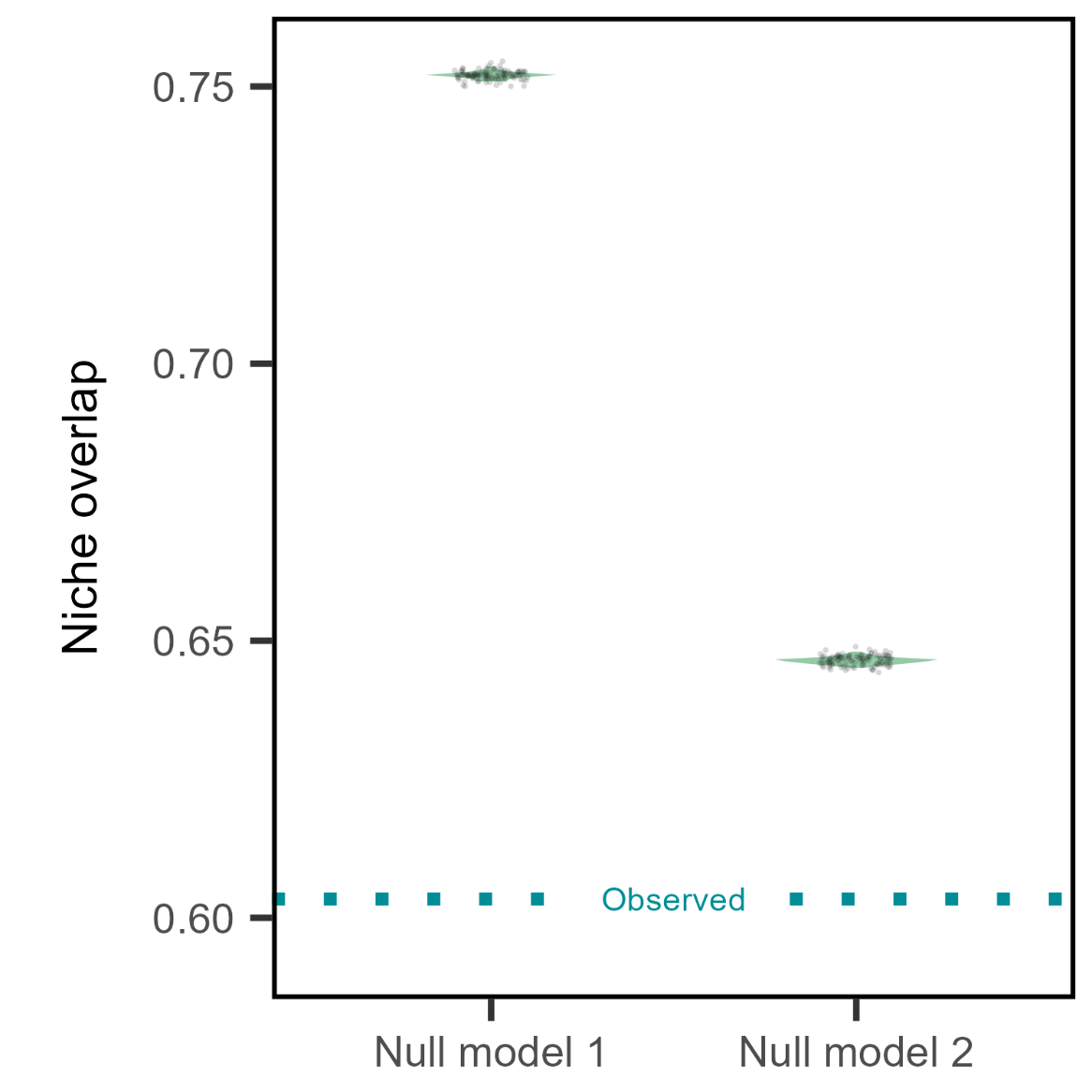


**Fig. S8.**

**
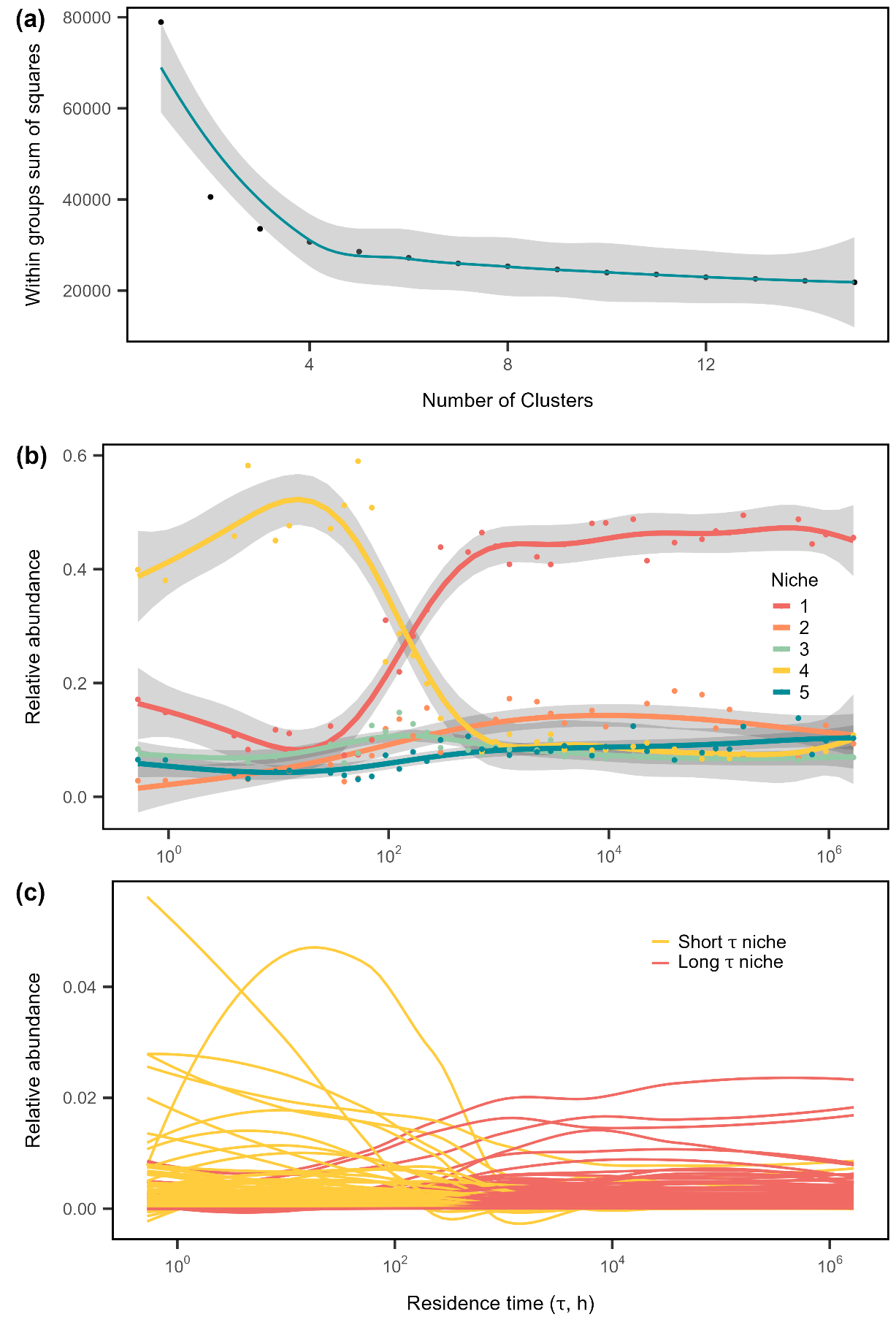
**

**Fig. S9.**

**
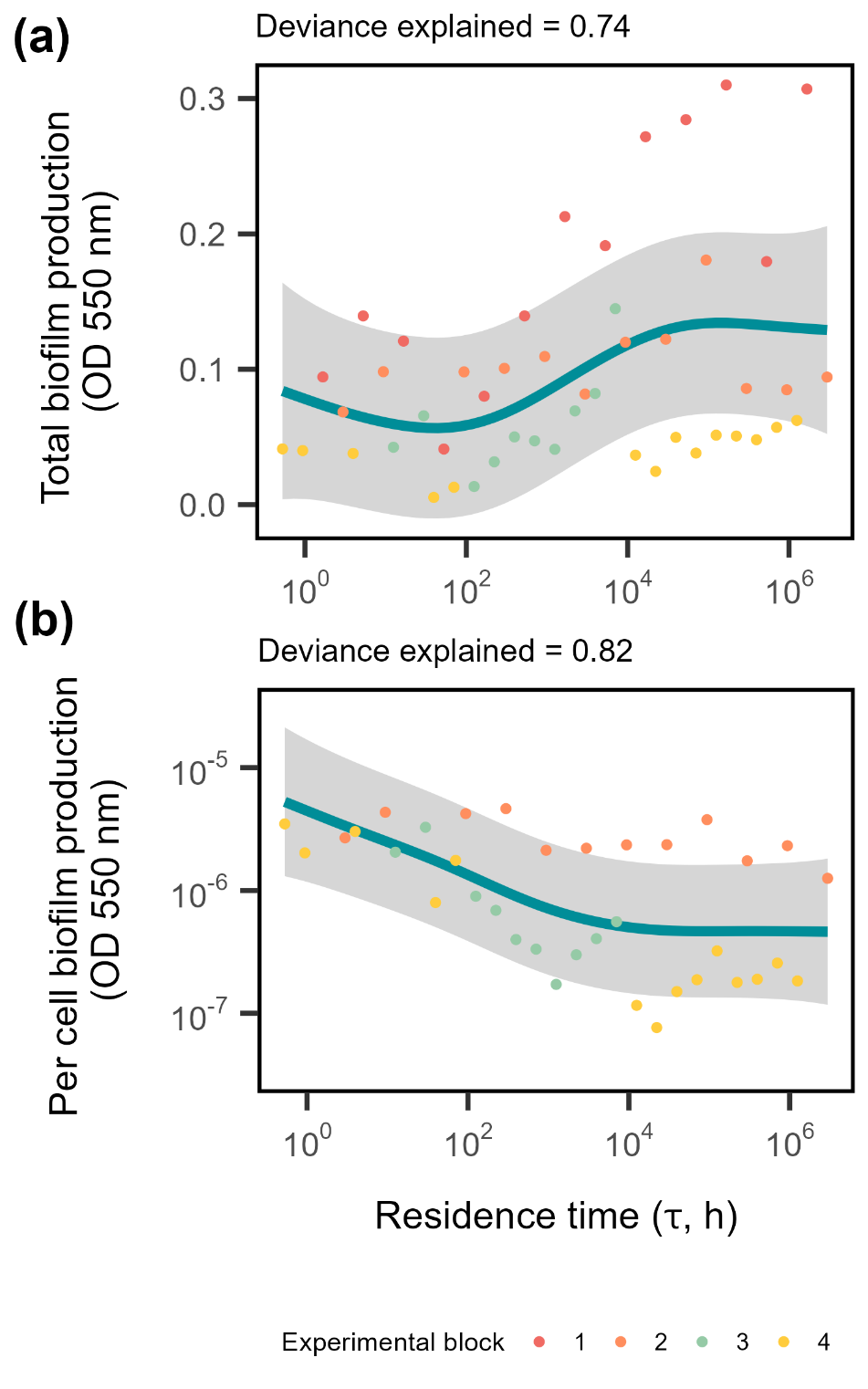
**
